# Supplementary material for: Interactions between the myosin Dachs, the adaptor Dlish, and the palmitoyltransferase Approximated mediate Fat-Dachsous signaling
Source: bioRxiv. 2026 Apr 16:2026.04.14.718308. Preprint. [Version 1] doi: 10.64898/2026.04.14.718308 (PMC13104975; doi:10.64898/2026.04.14.718308)
Supplement: 1 [file NIHPP2026.04.14.718308v1-supplement-1.pdf]

bioRxiv preprint doi: https://doi.org/10.64898/2026.04.14.718308; this version posted April 16, 2026. The copyright holder for this preprint (which was not certified by peer review) is the author/funder, who has granted bioRxiv a license to display the preprint in perpetuity. It is made available under aCC-BY 4.0 International license.

`

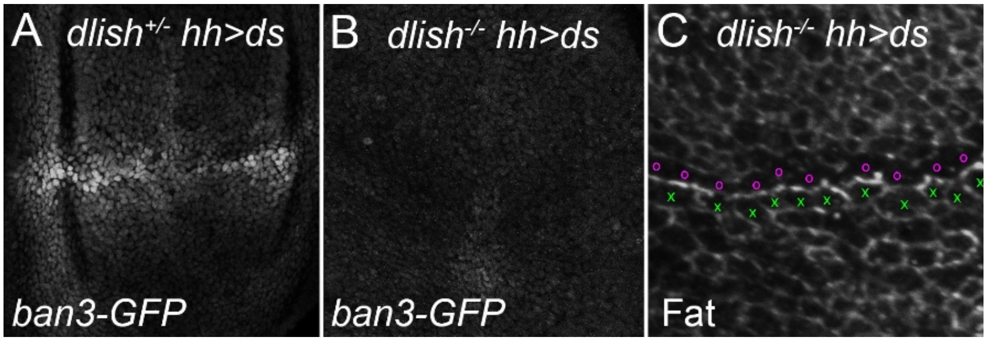

Figure S1. Effect of *dlish* loss on the activation of the Yki target *ban3-GFP* by boundaries of Ds overexpression in wing discs. A,B Expression of *ban3-GFP* after posterior, *hh-gal4-driven* expression of *UAS-ds* in wild type (A) and *dlish*^{B1601} homozygous (B) backgrounds. C. Depletion of anti-Fat staining from non-boundary cell faces (left and right) of cells just anterior to boundary (marked by magenta o) in *dlish*^{B1601} homozygote with *hh-gal4-driven* expression of *UAS-ds* (cells at anterior limit marked by green x*).*

bioRxiv preprint doi: https://doi.org/10.64898/2026.04.14.718308; this version posted April 16, 2026. The copyright holder for this preprint (which was not certified by peer review) is the author/funder, who has granted bioRxiv a license to display the preprint in perpetuity. It is made available under aCC-BY 4.0 International license.

`

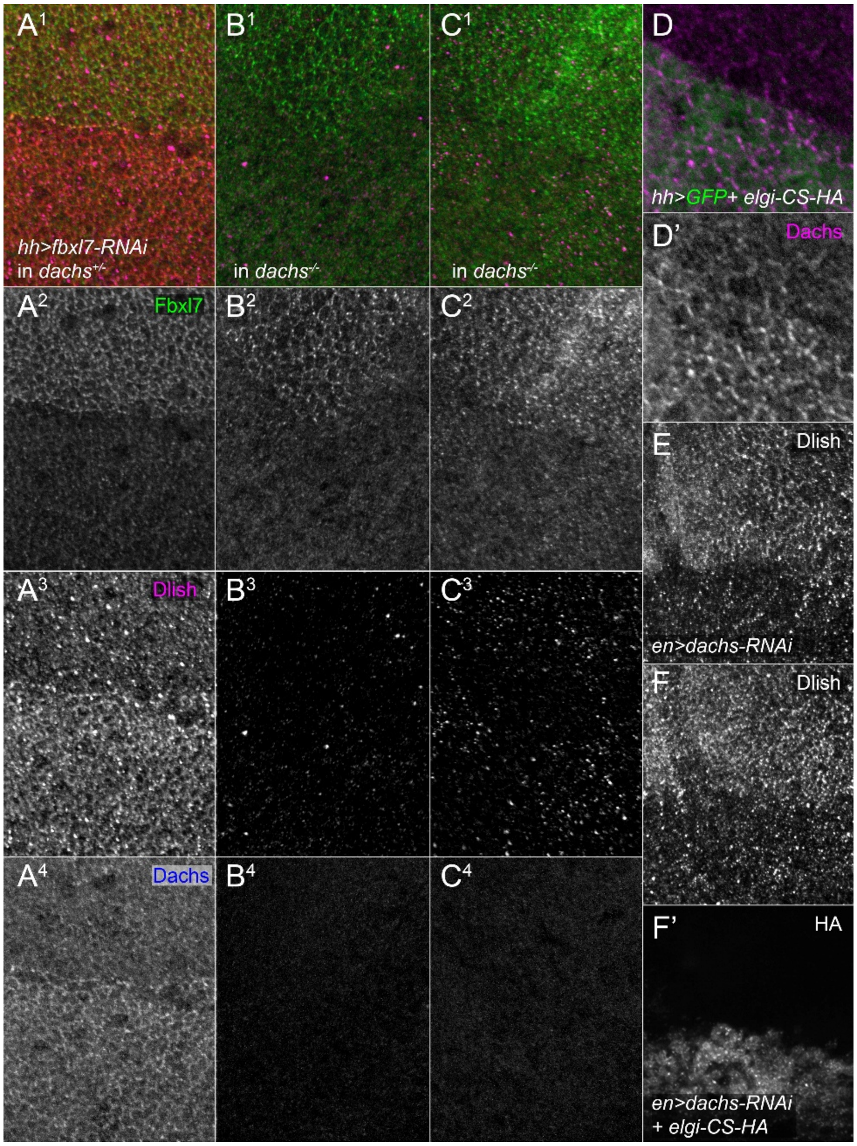

Figure S2. The effect of reducing of Fbxl7 or Elgi activity on the loss of Dlish caused by *dachs* loss or knockdown. A^1-C^4. Posterior, *hh-gal4*-driven expression of *UAS-fbxl7-RNAi* and its effect on anti-Fblx7 staining (green in A^1,B^1,C^1 and grey in A^2,B^2,C^2), anti-Dlish staining (magenta in A^1-C^1, grey in A^3-C^3), and anti-Dachs staining (blue in A^1-C^1, grey in A^4-C^4) in +/ *d^{210}* (A1-A4) or *d^{GC13}/d^{210}* wing discs (B^3,C^3) identified by the absence of anti-Dachs staining (B1^{-B4},C1-C4). D,D'. Anti-Dachs staining (magenta in D, grey in D') after posterior, *hh-gal4*-driven expression of the dominant negative *UAS-elgi-CS-HA*, identified by co-expression of *UAS-GFP* (green in D). E-F'. Anti-Dlish staining (E,F) after posterior, *en*-gal4-driven expression of U*AS-dachs-RNAi*,

bioRxiv preprint doi: https://doi.org/10.64898/2026.04.14.718308; this version posted April 16, 2026. The copyright holder for this preprint (which was not certified by peer review) is the author/funder, who has granted bioRxiv a license to display the preprint in perpetuity. It is made available under aCC-BY 4.0 International license.

`

without (E) or with{F) *UAS-elgi-CS-HA*, identified by anti-HA staining (F'). Discs being compared (those in A[1]-C[4], and those in E and F) were fixed and stained in the same well and imaged and processed with the same settings.

bioRxiv preprint doi: https://doi.org/10.64898/2026.04.14.718308; this version posted April 16, 2026. The copyright holder for this preprint (which was not certified by peer review) is the author/funder, who has granted bioRxiv a license to display the preprint in perpetuity. It is made available under aCC-BY 4.0 International license.

`

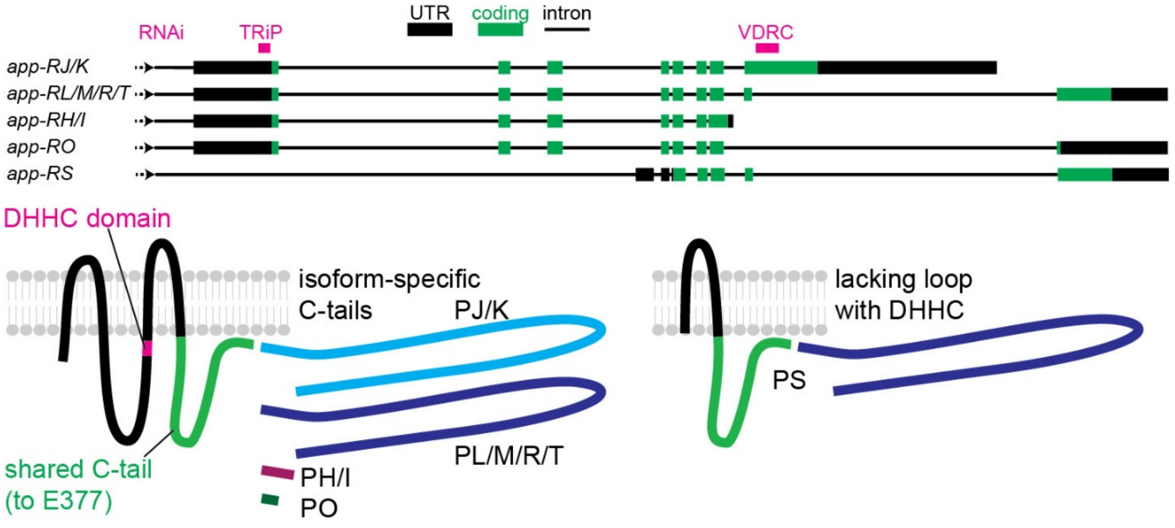

Fig. S3. Top: predicted alternative splicing of *app* transcript and targeting by the TRiP and VDRC *UAS-app-RNAi*'s. Bottom: predicted protein isoforms. Data from ref{Matakatsu, 2008 #757} and Flybase[62].

bioRxiv preprint doi: https://doi.org/10.64898/2026.04.14.718308; this version posted April 16, 2026. The copyright holder for this preprint (which was not certified by peer review) is the author/funder, who has granted bioRxiv a license to display the preprint in perpetuity. It is made available under aCC-BY 4.0 International license.

`

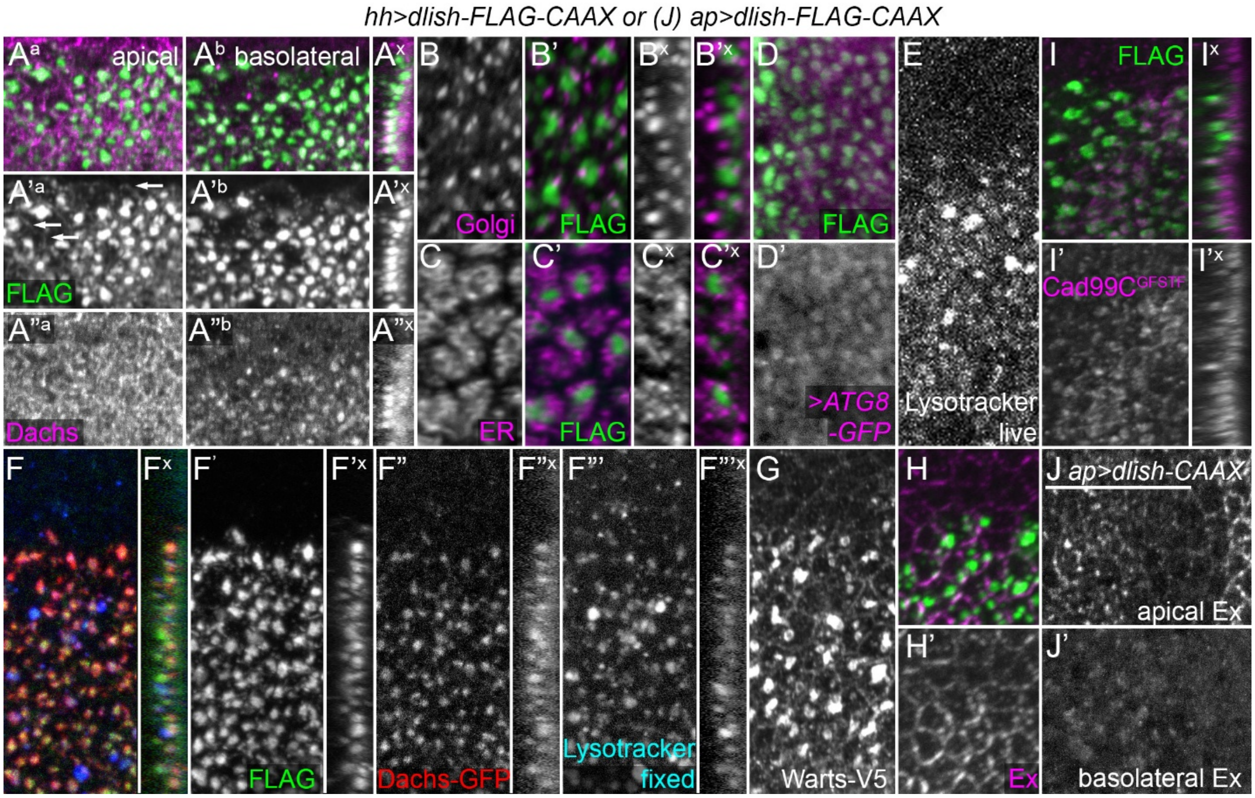

Figure S4. A-I. Colocalization of large anti-FLAG staining aggregates (green) after posterior, *hh-gal4*-driven expression of *UAS -dlish-FLAG-CAAX* with autolysosomal markers and other proteins. Panels marked with superscript x are cross-sections with apical to the right. A^{a}-A”^{x}. Anti-FLAG staining in membranes (A’^{a}, arrows) and aggregates; the latter partially overlap with anti-Dachs especially in slightly more basolateral sections (A^{b}-A”^{b}, cross-sections in A^{x}-A”^{x}). B-C’^{x}. Little overlap between FLAG aggregates and cell compartment markers EYFP-Golgi (B) or EYFP-ER (C), both expressed via *sqh*-derived regulatory sequences[63]. D,D’. Overlap between FLAG aggregates and *UAS*-driven ATG8-GFP. E. Posterior induction of Lysotracker staining of live discs. F-F”’^{x}. Partial overlap between FLAG aggregates, Dachs-GFP, and Lysotracker staining of live discs imaged after fixation. G. Induction of Warts-V5 aggregates. H,H’. No

bioRxiv preprint doi: https://doi.org/10.64898/2026.04.14.718308; this version posted April 16, 2026. The copyright holder for this preprint (which was not certified by peer review) is the author/funder, who has granted bioRxiv a license to display the preprint in perpetuity. It is made available under aCC-BY 4.0 International license.

`

accumulation of anti-Ex staining in FLAG aggregates. I,I'. Partial overlap of the GFP-tagged Cad99C^{GFSTF} protein trap with FLAG aggregates. J,J'. Weak accumulation of basolateral Ex aggregates after dorsal (left), *ap-gal4*-driven expression of *UAS-dlish-FLAG-CAAX*.

bioRxiv preprint doi: https://doi.org/10.64898/2026.04.14.718308; this version posted April 16, 2026. The copyright holder for this preprint (which was not certified by peer review) is the author/funder, who has granted bioRxiv a license to display the preprint in perpetuity. It is made available under aCC-BY 4.0 International license.

`

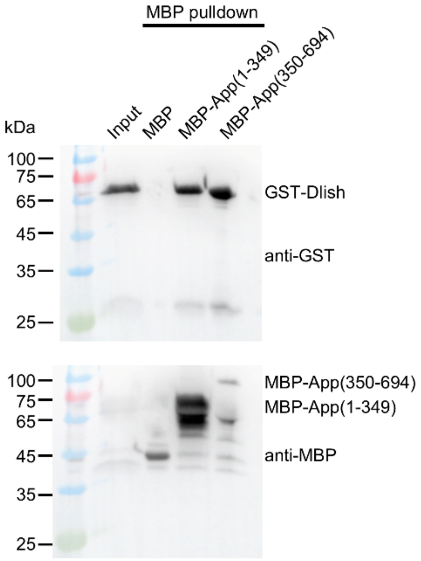

Figure S5. Pulldown of bacterially-produced GST-Dlish by bacterially produced MBP-App(1-349) or MBP-App(350-694).
